# Special Issue on Miniaturized Transistors, Volume II

**DOI:** 10.3390/mi13040603

**Published:** 2022-04-12

**Authors:** Lado Filipovic, Tibor Grasser

**Affiliations:** Institute for Microelectronics, TU Wien, Gußhausstraße 27-29/E360, 1040 Vienna, Austria

Due to the great success of the initial Special Issue on Miniaturized Transistors [1], we have decided to continue addressing the ever-advancing progress in microelectronic device scaling with this second volume. Complementary Metal-Oxide-Semiconductor (CMOS) devices continue to endure miniaturization, irrespective of seeming physical limitations to scaling, helped by advancing fabrication techniques. We also observe that miniaturization does not always refer to the latest technology node for digital transistors. Rather, by applying novel materials and device geometries, we note that a significant reduction in the size of microelectronic devices for a broad set of applications can be achieved. The achievements made in the scaling of devices for applications beyond digital logic (e.g., power applications, optoelectronics, and sensors) are taking the front stage in microelectronic miniaturization. Furthermore, all these achievements are assisted by improvements in the simulation and modeling of the involved materials and device structures. In particular, process and device technology computer-aided design (TCAD) has become indispensable in the design cycle of novel devices and technologies.

There are 19 research papers published in this Special Issue, covering recent advances in research aspects related to transistor miniaturization, including theoretical assessments of novel device geometries, the application of wide bandgap materials for high-power applications, modeling techniques for highly scaled devices, as well as devices for applications in optoelectronics and sensing. Furthermore, three reviews are included in this Special Issue, one which looks into transistor scaling along Moore’s Law and presents ideas about what lies ahead [2], another which investigates the applicability of metal oxide/polymer heterojunctions for flexible and portable electronics [3], and a third which performs an analysis of the reliability of highly scaled devices, where the charging kinetics of single defects play a critical role [4].

The three review articles which are provided in this Special Issue nicely summarize the impact of miniaturization on the current semiconductor device landscape. They include transistor scaling along Moore’s Law and the introduction of new materials and hetero-junctions for exciting new applications in flexible and portable electronics. However, miniaturisation and scaling come at a price, as is discussed in the third review, which looks into the impact of defects in highly scaled devices. Radamson et al. [2] provide a review on the development of the metal-oxide-semiconductor field-effect transistor (MOSFET) over the last decades while following the international technology roadmap of semiconductors (ITRS). They focus on methodologies, challenges, and difficulties when ITRS approaches the end and discuss new and emerging channel materials beyond the Moore era. Jeong et al. [3] provide a thorough review of the application of hybrid polymer/metal oxide films for flexible and wearable devices. These nanocomposites are excellent materials for flexible electronics due to the combined benefits of durability of the polymers and excellent electronic properties of the metal oxides. The authors highlight the advances made in improving the electrical performance of these devices by studying their mobilities and dielectric constants, as well as looking into interface engineering and its impact on the electronic properties. Waltl [4] wraps up this Special Issue with a thorough look into the reliability of highly scaled semiconductor transistors. As transistors are miniaturized further, single defects play an ever-increasing role in the device performance and reliability. Waltl takes a look into bias temperature instability (BTI) at the single-defect level to provide an in-depth investigation of charge trapping kinetics of the defects to ultimately provide an accurate assessment of the device lifetime.

The impact of single defects is also studied by Stampfer et al. [5]. The authors of this paper look at the adverse effects of miniaturization. Mainly, they observe how miniaturization leads to increased variability among nominally identical devices, primarily due to the increased relative impact of oxide traps. With random telegraph noise (RTN) measurements, the authors are able to extract the step heights of defects present at the Si/SiO_2_ interface. They note that, contrary to recently published studies, a bimodal distribution in step heights can be observed. Cheung, in [6], on the other hand, explores the potential application of a single-defect MOSFET device towards the realization of the elusive quantized current source. Their experimental results on a single-defect MOSFET shows that the one charge pumped per cycle is valid, encouraging further exploration of charge pumping based on quantum current sources.

With increased device scaling and the uncertainty of what lies beyond Moore’s law once the physical limits are reached, many researchers are theorizing about novel transistor designs. For this, applying advanced TCAD tools is indispensable. It is no longer a reasonable expectation that any potential design can be tested in a lab, as this comes with extremely high costs, especially at the leading technology nodes. Instead, many researchers rely on TCAD as an initial assessment of their proposed device ideas. With this in mind, in [7], Wulf presents a compact nanotransistor model which allows for the extraction of important device parameters such as the effective height of the source-drain barrier, device heating, and the quality of the coupling between the conduction channel and the contacts. This model is then used to quantitatively describe quantum transport in a variety of industrial nano-FETs. Medina-Bailon et al. [8] presented a quantum enhancement of a 2D Multi-Subband Ensemble Monte Carlo (MS-EMC) simulator, specifically to observe transistor behavior at the nanometer scale. Kim et al. [9] use TCAD simulations to examine the differences between the memory mechanisms in poly-silicon and silicon body one-transistor dynamic random-access memory (1T-DRAM) cells. They found that a poly-silicon 1T-DRAM can perform memory operations by using grain boundaries (GBs) as storage regions in thin body devices with a small floating body (FB) area. Zhang et al. [10] propose an implementation of a novel core-insulator gate-all-around (CIGAA) nanowire transistor which exhibits low off-state current compared to that of traditional gate-all-around (GAA) nanowire devices, making it ideal for future energy-efficient applications. Han et al. [11] proposed a germanium-based GAA transistor, which shows an all-around improved performance when compared to silicon GAA FETs. Specifically, the germainum GAA FET exhibits a higher ON/OFF ratio compared to silicon and a steady and steep average subthreshold swing. Chen et al. [12] presents a three-input, three-channel field effect transistor (TI-TcFET) design with multiple gate contacts (top, front, and back) in order to increase the gate control of the channel. The authors show that the proposed device could be used to simplify complex circuits by using less transistors than in traditional CMOS technology.

Beyond digital logic, high-power electronic devices are playing a very important role in today’s technology development. They are essential components in the push towards autonomous vehicles and safer air and space travel, while also providing important progress towards green energy applications and overall increase in energy efficiency. Six papers in this Special Issue look into novelties related to high-power electronic devices, mostly using wide-bandgap semiconductors such as silicon carbide (SiC) and gallium nitride (GaN). Chien et al. [13] study the application of a split-gate trench (SGT) power metal-oxide-semiconductor field-effect transistor (MOSFET) to reduce specific ON resistance. They note that the bottom epitaxial layer of a double-epitaxy structure can be designed to support the breakdown voltage, while the top one can be adjusted to reduce the ON resistance. Jia et al. [14] present a 4H-SiC metal-semiconductor field-effect transistor (MESFET) with layered doped and undoped regions. After optimizing the thickness of the undoped region, the authors obtained an increase in the power-added efficiency (PAE) of 85.8% and a saturation current increase of 27.4%, when compared to the double-recessed 4H-SiC MESFET. Li et al. [15] tested the surge reliability of 1200 V SiC MOSFETs from various manufactures by stressing them until failure. By decapping the failed devices and observing the cross-section of the damaged cell, they found that high temperature caused by excessive current flow through the devices during the surge tests is the main culprit for failure. In a contribution by Li et al. [16], the authors propose and test a SiC MOSFETs device which is able to meet the requirements of DC microgrid protection. Their prototype was developed, tested, and compared to the silicon-based insulated gate bipolar transistor (IGBT) alternative, showing high promise in its application in a solid-state circuit breaker. Guo et al. [17] looked into the thermal characteristics of pulse-operated AlGaN/GaN high-electron-mobility transistors (HEMTs). Their models show that the maximum channel temperature and thermal impedance of the devices are considerably influenced by the pulse width and power density, while the geometry of the gates, i.e., variations in the gate fingers and their width, have no effect on the channel temperature, as long as the total gate width and active area is kept constant. Zhu et al. [18] propose a new design for a multi-recessed double-recessed p-buffer layer 4H–SiC metal semiconductor field effect transistor (IMRD 4H-SiC MESFET) with high PAE. Their design shows an improvement in the PAE of almost 70%, when compared to similar state-of-the-art designs. In an additional contribution from Zhu et al. [19], a novel AlGaN/GaN HEMT is proposed, with a high gate and a multi-recessed buffer (HGMRB) for high-energy-efficiency applications. Their design promises an increase in the breakdown voltage by 16.7%, while the gate-to-source capacitance is decreased by 17%. The radio frequency (RF) simulations showed impressive PAEs of 90.8%, 89.3%, and 84.4% at 600 MHz, 1.2 GHz, and 2.4 GHz, respectively.

This Special Issue also looks at applications beyond digital logic and high-power applications. Several research papers have been included, which discuss novel sensing and imaging technologies. Cheng et al. [20] studied the potential of using vacuum channel transistors in low-loss and high-speed electronics for operation in high-temperature and high-radiation environments. Their measurements of vertical diodes further show that current and voltage vary based on the pressure and gas composition of the ambient, suggesting a potential application of these devices for gas and pressure sensing. Mao et al. [21] theorized a floating gate transistor with two control gates in order to provide active noise control in bio-electrical measurements. The advantage of their implementation is the ability to use a cost-efficient single-polysilicon CMOS fabrication process. Zhi et al. [22] presented a novel avalanche photodiode (APD) design, which is compatible with Taiwan Semiconductor Manufacturing Company (TSMC)’s standard CMOS process, realizing scaling in these devices by enabling an integration between the optoelectronic and digital components. The fabricated device is able to operate at a wavelength of 850 nm. Finally, McGhee and Georgiev in [23] apply semi-empirical density functional theory (DFT) calculations to study the surface transfer doping process between hydrogen-terminated (100) diamond and the metal oxides MoO_3_ and V_2_O_5_. Their study shows that both oxides act as electron acceptors and inject holes into the diamond structure, meaning that these metal oxides can be described as p-type doping materials for diamond. The study suggests the ability to use deposited metal oxides in an oxygen-rich atmosphere to enhance the surface transfer doping between diamond and the oxides.

Finally, we would like to take this opportunity to thank all the authors for submitting exceptional and highly relevant research papers to this Special Issue. We would also like to sincerely thank all the reviewers who took precious time to carefully examine and help improve the quality of all submitted manuscripts. Peer review is an essential component of good science, and they deserve recognition for the success of this Special Issue. It is our sincere hope that the results provided in this Special Issue prove useful to scientists and engineers who find themselves at the forefront of this rapidly evolving and broadening field. Now, more than ever, it is essential to look for solutions to find the next disrupting technologies which will allow for transistor miniaturization well beyond silicon’s physical limits and the current state-of-the-art. This requires a broad attack, including studies of novel and innovative designs as well as emerging materials which are becoming more application-specific than ever before.
